# Polarity transitions during neurogenesis and germinal zone exit in the developing central nervous system

**DOI:** 10.3389/fncel.2015.00062

**Published:** 2015-03-04

**Authors:** Shalini Singh, David J. Solecki

**Affiliations:** Department of Developmental Neurobiology, St. Jude Children’s Research HospitalMemphis, TN, USA

**Keywords:** neurogenesis, neuronal progenitor, neuroepithelial, neuronal polarity, cell junction, epithelial mesenchymal transition, delamination

## Abstract

During neural development, billions of neurons differentiate, polarize, migrate and form synapses in a precisely choreographed sequence. These precise developmental events are accompanied by discreet transitions in cellular polarity. While radial glial neural stem cells are highly polarized, transiently amplifying neural progenitors are less polarized after delaminating from their parental stem cell. Moreover, preceding their radial migration to a final laminar position neural progenitors re-adopt a polarized morphology before they embarking on their journey along a glial guide to the destination where they will fully mature. In this review, we will compare and contrast the key polarity transitions of cells derived from a neuroepithelium to the well-characterized polarity transitions that occur in true epithelia. We will highlight recent advances in the field that shows that neuronal progenitor delamination from germinal zone (GZ) niche shares similarities to an epithelial-mesenchymal transition. Moreover, studies in the cerebellum suggest the acquisition of radial migration and polarity in transiently amplifying neural progenitors share similarities to mesenchymal-epithelial transitions. Where applicable, we will compare and contrast the precise molecular mechanisms used by epithelial cells and neuronal progenitors to control plasticity in cell polarity during their distinct developmental programs.

## Introduction

**Figure 1:**
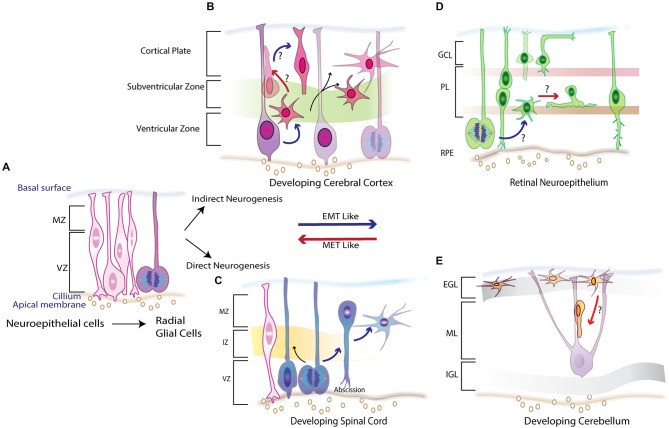
**Schematic overview of neurogenesis in the embryonic vertebrate CNS illustrating the various morphogenetic changes required to set up its laminar structure. (A)** Early neuroepithelium comprises of columnar cells that line the ventricular zone (VZ) and divide to give rise to Radial Glial Cells (RGCs) which proliferate giving rise to progenitors that make up different parts of CNS.**(B)** In the neocortex the proliferating RCGs set up a second proliferative zone called the sub ventricular zone (SVZ) undergo an indirect neurogenesis where one set of progeny inherits the cilliary component while the other adopts a multipolar morphology showcasing features of an EMT-like process. **(C)** In the developing spinal cord RGCs divide and by direct neurogenesis abscise their cilliary component, lose the adherens junctions (AJs) and adopt a multipolar morphology and migrate to intermediate zone (IZ) and marginal zone (MZ) recapitulating events that are similar to epithelial to mesenchymal (EMT) process. **(D)** Potential EMT-like and its reverse mesenchymal to epithelial transition (MET) are both displayed in the retinal pigmented neuroepithelium (RPE) where the newly born progeny frequently transitions between the two polarity states establishing and dissolving AJs to allow for migration to their correct spatial location in plexiform (PL) layer or the ganglion cell layer (GCL). **(E)** A MET-like parallel is observed in the developing cerebellum. External germinal layer (EGL) forms the second proliferative zone of neurons that arrive from the rhombic lip and possess a multipolar morphology with loss of any apical basal polarity. As development proceeded some of the epithelial like features are reacquired as they begin to migrate to inner granule layer (IGL) by riding the glial guided monorails in the molecular layer (ML). Red arrows denote these MET-like processes where the EMT–like process are depicted by blue arrows.

The developing central nervous system, including the spinal cord, retina and brain, occupies a complex developmental landscape wherein neural stem cells are born and then proliferate, differentiate and migrate long distances to form intricate networks, all of which ensure proper CNS functioning. Notably, almost all of the diverse cell types comprising the CNS originate from the neuroepithelium lining the embryonic neural tube and neural plate. Self-renewing multipotent cells (neural stem cells) orient themselves along the apical-basal axis in a single layer, conferring a highly polarized structure on this germinal niche. Subsequent symmetric and asymmetric division of the neural stem cells imparts a pseudostratified appearance to neuroepithelium, in which the nuclei undergo interkinetic nuclear migration while the apical-basal surfaces of the cells remain anchored through intercellular junctions (Figure 1A; Haubensak et al., 2004; Götz and Huttner, 2005). Alteration of polarity signaling cascade or cell adhesion dynamics leading to improper neural development substantiates the architectural organization of the neuroepithelium (Ayala et al., 2007; Métin et al., 2008; Roussel and Hatten, 2011). As development proceeds these progenitors must commit to a specific neuronal fate and migrate to their final destinations. This step requires them to sever ties with the ventricular zone (VZ), undergo a transition in polarity, change their adhesive preference and delaminate. Our understanding of key components and signaling cascades, such as the Par polarity complex and its interplay with adhesion molecules such as cadherins, nectins, claudins and junctional adhesion molecules (JAMs), has advanced considerably (Tsukita and Furuse, 1999; Mizoguchi et al., 2002; Costa et al., 2008; Ishiuchi et al., 2009; Famulski et al., 2010), but some key questions remain, including: What are the specific biological processes that precede delamination? What initiates and controls the switch in polarity and how is this linked to adherens junction (AJ) disassembly? Epithelial cells frequently display polar plasticity through processes known as epithelial-to-mesenchymal transition (EMT) and its reverse, mesenchymal-to-epithelial transition (MET), which developing neurons appear to mirror. By highlighting recent work addressing these specific challenges in the developing cortex, spinal cord, retina and cerebellum, we will highlight the emerging idea that akin to epithelial cells, progenitors in the GZs of the CNS require the EMT-MET machinery to undergo a change in polarity that leads to their delamination, differentiation and maturation.

## Developing neocortex

Development of the neocortex, with its unique lamination process, provides an attractive model to investigate the cellular remodeling essential for establishing the CNS (Rakic, 1971, 2009). Neural stem cells at the transient embryonic zone switch from a proliferative symmetric cell division phase to an asymmetric phase, giving rise to radial glial cells (RGCs), the progenitor cells of the cortex (Bayer, 1986, 1990; Malatesta et al., 2000; Campbell and Götz, 2002; Nadarajah and Parnavelas, 2002; Götz, 2003). Successive waves of migration at the VZ form an inside-out gradient of neurogenesis to establish the laminar cortical structure (Figure 1B; Angevine and Sidman, 1961). Stratification of the cortex requires radial and tangential migration of neurons, as shown by electron microscopy, lineage tracing and real-time imaging of brain slices (Mione et al., 1997; Wilson and Rubenstein, 2000; Marín and Rubenstein, 2001). Lamination of the cortex in CNS development requires precise spatial-temporal regulation of cortical migration (Métin et al., 2008). To guide the reader’s appreciation and understanding of the morphogenetic changes that occur during corticogenesis, we will briefly discuss the cytoarchitecture of the newly formed RGCs.

**Figure 2:**
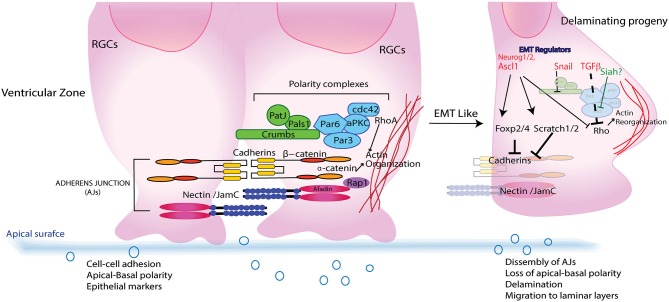
**A simplified schematic of subapical region of RGCs in VZ during CNS development**. RCGs resemble epithelial cells in having Adherence junctions (AJs) comprising of Cadherins and Nectins that intercalate and recruit polarity proteins like Crumbs and Par complex that imparts an apical–basal (AP) polarity to RGCs. Upon fate determination RCGs undergo cell division giving rise to daughter cells that delaminates and migrate away from the apical membrane to establish laminar layers in various parts of CNS. Epithelial to mesenchymal regulators such as transforming growth factor β (TGFβ), Scratch, Snail and Foxp2/4 that aid in polarity transition from an AP to a front–rear also interact with both structural (Cadherins) as well as signaling (Pard6-Rho/β catenin) components that leads to dissolution of AJs, reorganization of actin cytoskeleton and a morphological transformation that enables their journey to final destination.

Ultrastructure studies show that like epithelial cells, newly formed RGCs are morphologically polarized (Aaku-Saraste et al., 1996; Huttner and Brand, 1997; Chenn et al., 1998). They attach apically to the ventricular surface while extending long basal processes that span the entire cortical plate that affix at the overlying matrix produced by the pia. Apical anchoring is mediated by specialized intercellular adhesion complexes that involve cadherins, nectins, JAMs and β-catenin (Aaku-Saraste et al., 1996; Zhadanov et al., 1999; Manabe et al., 2002; Junghans et al., 2005; Kadowaki et al., 2007). These complexes link the cytoskeletal scaffolds and coordinate signaling pathways in neighboring cells while the pial attachment is established by integrins and possibly cadherins (Anton et al., 1999; Graus-Porta et al., 2001). Relevance of the cell-cell contact in establishing the radial glial scaffold at the apical surface and its role in signaling networks such as reelin is highlighted by cortical defects observed in β1integrin and Dab1/Rap1/cadherin deficient mice (Graus-Porta et al., 2001; Franco et al., 2011). As in epithelial cells, Cdc42 and polarity complexes such as Par proteins and Crumbs complex (Crb, PALS, PATJ, Lin7) are essential in establishing and maintaining these AJs (Figure 2; Manabe et al., 2002; Cappello et al., 2006; Imai et al., 2006; Bultje et al., 2009). Nevertheless, as development proceeds the RGCs divide giving rise to a progeny that become committed to their fate and must migrate to occupy their respective positions in the cortical layers. It appears that the newly born neurons must first break their AJs to delaminate from the apical surface. Second, they must remodel their cytoskeletons to initiate movement out of the VZ. Remodeling of polarity is apparent from time-lapse imaging and electron microscopy that show migrating neurons adopting an intermediate multipolar state before reacquiring some of the features needed for glial–guided migration (Shoukimas and Hinds, 1978; Nadarajah et al., 2003). Remodeling of junctions and polarity in these newly born neurons closely resemble the sequence of events during EMT-MET in epithelial cells (Figure 1B; Nelson, 2009; Lamouille et al., 2014).

Given that apical anchoring and delamination are crucial to cortical arrangement there has been great focus on understanding the apical protein complexes that allow for this GZ exit; and have helped to define the roles of N-cadherin/Rap1, adaptor protein Afadin, Lis1, β-catenin, cdc42/GSK-3β and polarity protein Pard3/Notch in maintaining the apical cytoarchitecture (Bos et al., 2001; Ooshio et al., 2007; Bi et al., 2009; Severson et al., 2009; Yokota et al., 2010; Franco et al., 2011; Jossin, 2011; Jossin and Cooper, 2011). A recent report showed that cadherin-based adhesions also facilitate Notch signaling (Hatakeyama et al., 2014). While these studies have identified key components of the machinery involved in establishing the polarity and GZ occupancy, the overarching question of how these components might be regulated demands attention. Because fate commitment and delamination are sequential events, it seemed plausible that proneural genes might also have a role in regulating delamination. Actin reorganization by proneural factors such as Neurogenin 2 (Neurog2) and Ascl1, activating Rho GTPases Rnd2 and Rnd3, respectively, allows remodeling of the actin cytoskeleton by inhibiting Rho activity, thus linking neuronal commitment and migration (Ge et al., 2006; Heng et al., 2008; Pacary et al., 2011). Although they may provide the physical force needed for RGC exit from the GZ niche, they do not initiate delamination, as silencing of Rnd3 disrupts the distribution of β-catenin and N-cadherin at AJs, indicating that Rnd3 is essential for maintaining the integrity of the junctions (Figure 2; Pacary et al., 2011, 2013). Additionally, the physical forces can be effective only if the RGCs first attenuate their intercellular adhesion through dissolution of AJs, lose their apical-basal polarity and acquire a multipolar nature. In neural crest cells and epithelial cells, this phenomenon is regulated by EMT factors that promote loss of epithelial characteristics and acquisition of mesenchymal attributes (Thiery and Sleeman, 2006; Baum et al., 2008). Central to this process are the Snail, Slug and Zeb family of transcriptional repressors (Chaffer et al., 2007; Baum et al., 2008). From these systems and the fact that newly committed neurons express such regulators, one can extrapolate a possible molecular link between commitment and delamination.

In support of the above idea, Scratch 1 and Scratch 2, members of the Snail superfamily, are expressed upon neuronal fate commitment in neocortex, in which they trigger active disintegration of AJs by directly repressing expression of E-cadherin (Figure 2; Itoh et al., 2013). Additionally, classical genes in the EMT pathway, such as those encoding Occludin and Nephrins, also show downregulation accompanied by increased expression of Mmp19 (Thiery and Sleeman, 2006; Itoh et al., 2013). Notably, Scratch1 overexpression has no effect on neurogenesis, thus identifying it as a delamination-specific pathway. The existence of an EMT-like pathway in corticogenesis is further buttressed by the finding that another regulator of the pathway, Snail, is expressed in both radial precursors and newborn neurons during corticogenesis (Zander et al., 2014a,b). Snail’s role in the neocortex is not limited to regulating AJs; it also helps promote the survival, proliferation and self-renewal of cortical progenitors.

## Developing spinal cord

The central question of what molecular mechanism controls AJ disassembly during migration has been addressed in developing motor neurons (MNs) of the spinal cord. Spinal cord development is a multistage process with distinct subtypes V0, V1, V2, V3, interneurons and MNs (Figure 1C; Alaynick et al., 2011). Convergent action of morphogens such as Shh and retinoic acid elicits a unique set of transcriptional networks and factors (e.g., Olig2) that specify different neuronal subtypes (Mukouyama et al., 2006; Briscoe and Novitch, 2008). Studies in Olig2-mutant mice showed that the forkhead proteins Foxp1, 2 and 4 are essential for specifying MN fate and for migration (Ferland et al., 2003; Dasen et al., 2008; Rousso et al., 2008; Palmesino et al., 2010). Rousso et al. investigated the role of Foxp proteins in chicken and mouse spinal cord, showing them to be components of a gene regulatory network that links and balances AJs with cell fate (Rousso et al., 2012). Increased expression of Foxp2 and Foxp4 inversely affected expression of N-cadherin and Sox2, leading to dissolution of AJs and ectopic neurogenesis in the VZ. Conversely, their loss maintained the precursors in the progenitor state (Figure 2). Such transcriptional regulation of N-cadherin and Sox2 might also regulate the size of the embryonic VZ niche by controlling delamination (Rousso et al., 2012). Decreased Sox2 and cadherin levels have also been demonstrated in neural crest cells before delamination (Zappone et al., 2000; Bylund et al., 2003; Graham et al., 2003; Bello et al., 2012). These findings, together with the documented role of forkhead proteins in maintaining AJs in other systems by regulating cadherins, reinforces the idea of an EMT-like signature in CNS development (Figure 1C; Mani et al., 2007; Rousso et al., 2012).

Besides transcriptional control of adhesion proteins, delamination of progenitors also involves a change in polarity and cellular architecture, recently addressed in a new light by Das and Storey. By using structured illumination microscopy in chick neural tube, they discovered a cellular mechanism called apical abscission that participates in actin-myosin–dependent remodeling of primary cilium (Das and Storey, 2014). Apical abscission at the VZ detaches progenitors and leads to loss of apical complex proteins, a process characterizing loss of apical polarity (Farkas and Huttner, 2008; Das and Storey, 2012). This study answered the question of how the VZ, unlike archetypal epithelium undergoing EMT, maintains its integrity. It seems plausible that apical abscission might provide the progenitors an efficient way to transiently change their polarity without substantially altering their transcriptional profiles. If basal progenitors remodel their primary cilia in preparation for delamination in the neocortex and for apical shedding of MNs that could explain how newly differentiated neurons lose and reacquire polarity during delamination and migration by remodeling their apical complex proteins.

## Developing retina

Like other developing CNS structures, the retinal neuroepithelium is pseudostratified. During retinogenesis, polarized progenitors remain anchored to the apical-basal membrane by AJs and TJs while their fate is determined by interkinetic nuclear migration (Figure 1D; Frade, 2002; Baye and Link, 2007, 2008). Subsequent establishment of the retinal layers requires post-mitotic detachment of retinal progenitors from the apical surface, reorganization of polarity and migration to the appropriate layers (Miyata, 2008).

In mouse retinal progenitors, polarity is imparted by two apical complexes, the partitioning defect (PARD) complex comprising Pard3, Pard6, aPKC and cdc42 and crumbs-homologue (CRB) comprising Crb1-3, Pals1/MPP5 and Patj (Koike et al., 2005; Cappello et al., 2006; van de Pavert et al., 2007; Park et al., 2011; Alves et al., 2013; Dudok et al., 2013; Heynen et al., 2013). In mice lacking CRB2, early retinal progenitors show abnormal lamination due to greater proliferation of last-born progenitors. Membrane-palmitoylated protein 3 (MPP3), a scaffolding cell-cell adhesion protein, is also localized to the retinal sub-apical region and interacts with Pals1 and Dlg1 (Pellikka et al., 2002; Alves et al., 2013). Perturbation of MPP3 in developing retina was found to cause focal disruption of AJs and ectopic cell localization (Dudok et al., 2013). The need to maintain polarity is further stressed by laminar defects in Pals1 conditional-knockout mice, in which mislocalization of neurons was found throughout the retina (Park et al., 2011). Additionally, dysregulation of aPKC and cdc42 lead to lamination defects (Koike et al., 2005; Heynen et al., 2013).

Structural and signaling components come together at neural junctions. In vertebrates, the polarized epithelium’s apical junction proteins, such as cadherin, occludins, claudins and JAMs, interact on the cytoplasmic side of the cell with a variety of polarity and signaling molecules, including Pard3 and β-catenin, that connect them to the actin cytoskeleton (Tsukita and Furuse, 1999; Tsukita et al., 1999; Nishimura et al., 2004; Umeda et al., 2004; Perez-Moreno and Fuchs, 2006). In the retina, alteration of β-catenin causes loss of polarity and adhesion, cell detachment, ectopic migration and spatiotemporally perturbed retinogenesis (Brault et al., 2001; Fu et al., 2006). The role of apical complexes is exemplified in retinogenesis, in which they not only establish adhesion and polarity but act as important nodes for diverse signaling pathways (e.g., Notch, Wnt, Hippo, mTOR) essential for specifying retinogenesis. As supporting evidence, Ohata et al., showed that Crb complex and Notch form a positive feedback loop that maintains apicobasal polarity; disruption of feedback causes laminar defects (Ohata et al., 2011).

There are some interesting differences between retinal neuroepithelium and other developing CNS structures, one of which was highlighted by Ivanovitch et al. By combining mosaic labeling of single cells with 4D confocal imaging during optic evagination in zebrafish, they showed two discrete populations of cells: basally positioned cells and core cells involved in establishing the retinal neuroepithelium (Ivanovitch et al., 2013). Interestingly, live imaging showed that basally positioned cells undergo precocious polarization, while core cells remain mesenchymal until optic vesicle formation. The authors found that core cells that localize Pard3 in a polarized manner undergo a special MET using apical points for intercalation in CNS outpocketing. Furthermore, modulation of Pard6γb and Laminin leads to failure of optic vesicle evagination (Ivanovitch et al., 2013).

Clearly, the transition in polarity and morphology of retinal progenitors requires rearrangement of their cytoskeleton that generates physical forces required for movement. While this process is a quintessential requirement for determining the laminar structure of developing CNS, how are such processes regulated remains an open question. A chemical mutagenesis screen in medaka fish identified the guanine-nucleotide exchange factor ArhGEF18, a RhoA- and Rac-specific GEF factor, to be a key regulator of retinal architecture and function that also controls apicobasal polarity and proliferation (Herder et al., 2013; Loosli, 2013). In epithelial cells, small GTPases of the Rho family act as molecular switches that regulate and coordinate the actin cytoskeleton, cell junction assembly and polarity (Heasman and Ridley, 2008; Samarin and Nusrat, 2009). Additionally, Rho GTPases are regulated by transforming growth factor β (TGFβ), which degrades RhoA by phosphorylating Pard6 (Ozdamar et al., 2005). TGFβ also downregulates Pard3 expression while activating the EMT regulator Snail (Figure 2; Wang et al., 2008). As TGFβ is expressed during early retinal embryogenesis, it could plausibly play a role in regulating or guiding a similar process at that time.

## Developing cerebellum

The cerebellum is an attractive model for studies of CNS development, primarily because of its remarkably simple laminar organization, which consists of two principal neurons (cerebellar granule neurons (CGNs) and Purkinje cells) and minority interneuronal populations (Roussel and Hatten, 2011). Granule neuron progenitors (GNPs) arise along the rhombic lip, at the midbrain-hindbrain junction, and migrate dorsally over the cerebellar anlage (Wingate, 2001), while a stream of radially migrating progenitors arising from neuroepithelium form the other cerebellar cell types (Hallonet and Le Douarin, 1993; Gao and Hatten, 1994). After GNPs migrate away from the rhombic lip, they proliferate under the guidance of external cues, such as Shh, to generate a second GZ called the external granular layer (EGL; Hatten, 1999). The remaining cerebellar histogenesis occurs mainly postnatally. This protracted development provides an excellent opportunity to study distinct intermediate stages of neurogenesis. After their proliferative phase, GNPs exit the cell cycle to form a layer of cells that migrate tangentially, then undergo a morphological transformation that initiates radial migration through the molecular layer (ML) along glial fibers to their final laminar position (Rakic, 1971; Edmondson and Hatten, 1987; Komuro et al., 2001). In the outer EGL, the GNPs have a round morphology with multiple short processes (Manzini et al., 2006). As they migrate to the inner EGL, their cytoskeleton is reorganized to form two short horizontal processes that elongate during their journey to the ML (Figure 1E). After a brief latency at the ML, they acquire an elongated spindle shape and a vertical process (Komuro et al., 2001).

The maturation of CGNs and their migration requires polarization, which was carefully examined in two studies delineating role of Par complex in this process. The first study showed that manipulation of Pard6α function, both *in vitro* and *ex vivo*, disrupts the actomyosin cytoskeleton and blocks radial migration of CGNs (Solecki et al., 2009). As expression of Pard3A polarity protein increases as the CGNs mature, the second study investigated its role in CGN migration (Famulski et al., 2010). Whereas loss of Pard3A impeded radial migration of CGNs, ectopic expression led to precocious migration. Further, Pard3A expression was shown to be regulated by E3 ubiquitin ligase seven in absentia homolog (Siah) 1 and 2 (Figure 2). Reciprocal expression of Siah and Pard3A in developing CGNs indicated that Siah negatively regulates Pard3A expression. This study also linked polarity proteins with adhesion of CGNs. In the epithelial cells, Pard3A binds to three members of the JAM family via its PDZ domain and recruits them to TJs to establish polarity (Ooshio et al., 2007). Utilizing live probe imaging, Famulski et al., demonstrated identical spatiotemporal expression of these proteins during CGN maturation and showed that JAM-C is necessary and sufficient for CGN exit from the EGL (Famulski et al., 2010). These results indicate that not only is cell adhesion crucial for guiding a neuron’s migration path but the acquisition of adhesion by CGNs and loss of some of multipolar features reflects the MET-like process (Figure 1E). This is unique from other developing CNS structures where the newly born neurons lose polarity and require it upon reaching their final laminar position. A pertinent question that sequentially arises from these observations is how polarity is reorganized and how immature neurons initiate, implement and conclude this essential morphogenic event.

## Summary

Addressing the morphological changes observed in newly born neurons and its relevance in setting up the laminar structure of CNS raises some interesting questions. How the polar plasticity of newly born is regulated and what are the key factors that are involved in this process across the various developing CNS structures? Further in the developing cerebellum, how do the neurons born from the RGCs after adopting a multipolar migratory feature upon reaching the second GZ restore their polarity? Like the epithelial system is there a regulator whose expression temporally coincides with the CGNs reacquiring epithelial characteristics aka MET like processes. If so, what would be some of the targets of this MET like processes in developing neurons? Do these factors also play a role in differentiation and maturation of the neurons?

## Conflict of interest statement

The authors declare that the research was conducted in the absence of any commercial or financial relationships that could be construed as a potential conflict of interest.
